# Camouflaging in Autism: Age Effects and Cross-Cultural Validation of the Camouflaging Autistic Traits Questionnaire (CAT-Q)

**DOI:** 10.1007/s10803-023-05909-8

**Published:** 2023-02-09

**Authors:** Karl Lundin Remnélius, Sven Bölte

**Affiliations:** 1 Center of Neurodevelopmental Disorders (KIND), Centre for Psychiatry Research, Department of Women’s and Children’s Health, Karolinska Institutet & Stockholm Health Care Services, Region Stockholm, Gävlegatan 22B, 113 30, Stockholm, Sweden; 2 Child and Adolescent Psychiatry, Stockholm Health Care Services, Region Stockholm, Stockholm, Sweden; 3 Curtin Autism Research Group, Curtin School of Allied Health, Curtin University, Perth, WA Australia

**Keywords:** Autism, Camouflaging, Validation, Gender differences, Trajectories

## Abstract

Given that camouflaging has been suggested to contribute to delayed diagnosis and mental health problems among autistic people, validated measures of the construct are needed. This study describes the psychometric evaluation of the Swedish adaptation of the self-reported Camouflaging Autistic Traits Questionnaire (CAT-Q) in autistic (*n* = 100) and general population (*n* = 539) samples aged 10 to 83 years. Analyses indicated good-to-excellent internal consistency and test-retest reliability. Construct validity was supported by autistic participants scoring higher than non-autistic, and autistic females scoring higher than autistic males on the measure. Also as expected, camouflaging was associated with theoretically linked traits, including autistic behaviors and depressive symptoms. On the other hand, the factor structure of the original CAT-Q did not provide a good fit in the Swedish data set, and validity issues were found, particularly in children younger than 15 years, warranting further investigation of the construct validity of the scale. Different age trajectories were observed, where camouflaging behaviors decreased during adulthood in non-autistic people but remained at an elevated level among autistic people.

**Supplementary Information:**

The online version contains supplementary material available at 10.1007/s10803-023-05909-8.

## Introduction

Research suggests that autistic people might apply strategies contributing to “less autistic” behavior presentations in social interaction, so called *camouflaging* (Hull et al., 2018). Such strategies may include suppressing repetitive behaviors, effortfully making eye contact, or having prepared “scripts” for conversations (Hull et al., 2017). Autistic people describe that camouflaging strategies are motivated both by external demands for being accepted in society and avoid retaliation and by internal motivation to socialize and develop relationships (Cage & Troxell-Whitman, 2019; Hull et al., 2017; Tierney et al., 2016).

Some camouflaging behaviors might tap compensatory cognitive resources such as executive functions to explicitly plan socially accepted behavior or inhibit undesired behaviors, instead of relying on implicit social cognition resources, which are commonly altered in autism (Livingston & Happé, 2017). Such cognitive resources are limited and compete with nonsocial cognitive demands, making camouflaging behaviors effortful and tiring (Livingston & Happé, 2017). Consistently, autistic people describe that camouflaging is linked to negative consequences, including exhaustion, anxiety, and stress (Bargiela et al., 2016; Cage et al., 2018), which has been supported by quantitative research reporting associations between camouflaging and detrimental outcomes, including mental health problems and suicidality (Cage & Troxell-Whitman, 2019; Cassidy et al., 2018; Hull et al., 2021).

Camouflaging behaviors are not exclusively reported in autistic people, but also among non-autistic individuals, albeit to a lesser extent (Dell’Osso et al., 2022; Hull et al., 2018). Therefore, the construct validity of camouflaging has been debated, particularly whether the construct is specifically related to autism or also reflect similar behaviors in other conditions, such as social anxiety disorder (Fombonne, 2020). In the literature, the presence of camouflaging among non-autistic people has been attributed to varying levels of autistic traits distributed across the general population (Hull et al., 2018). Another possibility is that camouflaging conceptually overlaps with ubiquitous human attempts to control other people’s impression of them, termed *impression management* (Ai et al., 2022; Goffman, 1959). Interestingly, previous research has indicated that autistic people might be less concerned than neurotypical people about presenting themselves in ways that elicit positive attitudes in others, i.e., managing reputation (Chevallier et al., 2012; Izuma et al., 2011), seemingly contrasting the notion of increased camouflaging in autistic people. Considering the above, further investigations into the construct validity of camouflaging, and its operationalizations are required.

## Moderators of Camouflaging

Previous studies indicate that camouflaging may be moderated by demographic factors, including sex/gender and age. In the autistic population, camouflaging has been suggested to be more common among girls and women (Lai & Baron-Cohen, 2015), potentially influenced by differences in social demands placed on females compared to males (Bargiela et al., 2016). Research evidence supports that autistic females use camouflaging more extensively compared to autistic males (Cola et al., 2020; Dean et al., 2017; Hull et al., 2019; Lai et al., 2017). On the other hand, a few studies comparing non-autistic females and males failed to find differences, suggesting that sex/gender differences in camouflaging are likely specific to autism (Hull et al., 2019). Two studies including autistic people identifying as non-binary found no significant differences in camouflaging compared to males or females (Hull et al., 2019; Perry et al., 2022).

Age might also influence camouflaging, where social maturation, social experiences, and formation of identity could contribute to age-related changes in camouflaging (Jorgenson et al., 2020). Camouflaging behaviors have been reported among autistic children (Dean et al., 2017), adolescents (Jedrzejewska & Dewey, 2022; Tierney et al., 2016), and adults (Bargiela et al., 2016; Hull et al., 2018). However, there are limited data on how these behaviors change with increasing age. Recent research implies an age effect on camouflaging, that might differ between autistic and non-autistic people. Autistic adults generally report higher camouflaging levels compared to non-autistic adults (Cook et al., 2021), whereas the few studies including younger participants have yielded mixed findings. Jorgenson and colleagues (2020) found no difference in overall camouflaging between autistic and non-autistic adolescents, whereas Jedrzejewska and Dewey (2022) observed more camouflaging among autistic participants. Still, the autistic adolescents in the latter study had a lower mean score on the Camouflaging Autistic Traits Questionnaire (CAT-Q) than what has been reported for adults on the autism spectrum using the same measure. The above findings could imply that camouflaging in autism follows a trajectory where use of such strategies increase during late adolescence and young adulthood, which might differ from the development of similar behaviors in non-autistic people.

## Measuring Camouflaging

Different operationalizations of camouflaging have been used in the literature, where some studies utilized the discrepancy between “internal” autism status, i.e., self-reported autistic traits or social cognition ability, and “external” presentation, often autism diagnosis defining social communication symptoms measured by the Autism Diagnostic Observation Schedule (ADOS). In this discrepancy approach, an internal-external mismatch indicated by a profile of high self-reported autistic traits or low scores on tests of social cognition in combination with low clinician-rated autistic traits/symptoms, is used as a proxy measure to indicate camouflaging (Lai et al., 2017; Livingston, Colvert, et al., 2019; Rynkiewicz et al., 2016).

In order to assess the first-person perspective, Hull and colleagues (2018) developed a self-report measure of camouflaging, the CAT-Q. The questionnaire aims to pick up behaviors and intentions, including “unsuccessful” attempts of camouflaging, which would be missed using the discrepancy approach (Hull et al., 2018). While the original version of the CAT-Q has demonstrated promising psychometric properties, other scales used in autism assessment have shown intercultural differences in standardization even between high-income countries presumed to be relatively comparable, such as the USA and countries in Europe (Bölte et al., 2008), and a lack of culturally appropriate validation of instruments in autism has been noted (Bölte et al., 2018).

As stated above, camouflaging has been reported among children, adolescents, and adults. Still, the CAT-Q has not been validated for children and adolescents, and previous studies have largely used discrepancy methods and parent-report to measure camouflaging in children (Cook et al., 2021). This is particularly unfortunate, as camouflaging has been argued to be elevated in autistic girls, contributing to later identification (Bargiela et al., 2016; Lai & Baron-Cohen, 2015), and a psychometrically sound measure of camouflaging in childhood might support timely autism recognition in young females. Moreover, the risk of mental health problems is increased in autism; co-occurring psychiatric conditions are common (Lai et al., 2019; Simonoff et al., 2008), and autistic people are at heightened risk for suicidal behaviors and suicide (Hirvikoski et al., 2016, 2019). As previous studies suggest camouflaging as a candidate risk factor for psychiatric symptoms and reduced quality of life, further research into the construct and validation of camouflaging measures are warranted.

## Aims

In order to provide a valid camouflaging measure for use in research and clinical services in Sweden, this study aimed to (i) investigate the psychometric properties of the Swedish version of the CAT-Q (henceforth denoted as the CAT-Q/SE) including the utility in children aged 10 to 15 years, and (ii) explore self-reported camouflaging levels across age groups in the autistic and the general population samples separately.

Based on previous evidence, we hypothesized that autistic participants would score higher on the CAT-Q/SE than non-autistic participants from the general population, that autistic females would score higher than autistic males, and that the CAT-Q/SE scores would be significantly associated to theoretically linked traits, including autistic traits and depressive symptoms.

## Methods

### Participants

The final sample included N = 639 participants, comprising 100 autistic individuals and 539 from the general population. Thirteen participants (10 from the general population samples and 3 autistic participants) were a priori excluded due to straight lining throughout the questionnaires. The autistic participants (55% identifying as female) were aged between 10 and 68 years (*M* = 28.3 years, *SD* = 14.9). A larger proportion of females compared to males was sought to reflect the likely target population for CAT-Q/SE, based on research suggesting higher propensity of camouflaging in autistic females (Cook et al., 2021). In the autistic sample, 9% reported being diagnosed with autism in early childhood (0 to 7 years), 28% in middle childhood (7 to 13 years), 12% in adolescence (13 to18 years) and 50% in adulthood (> 18 years). One autistic participant did not report age when diagnosed. Among autistic participants, 50% reported having been diagnosed with a co-occurring psychiatric condition (e.g., anxiety or bipolar disorder), 41% reported ADHD, 5% intellectual disability (ID), 7% other neurodevelopmental conditions (e.g., a specific learning or language disorder). Note that participants can be included in more than one of the diagnostic categories, e.g., having a psychiatric condition and ADHD. Regarding occupation, 39% of autistic participants reported that they were studying, 18% working, 11% being retired, 11% being on sick leave, and 10% unemployed. The remainder (11%) described another occupation in free text, for example being in work training or job-seeking.

In the general population group (61% identifying as female), participants were aged between 10 and 83 years (*M* = 34.6, *SD* = 18.7). Among these participants, 21% reported a lifetime diagnosis of a psychiatric condition (e.g., depression or an eating disorder), 9% ADHD, 0.6% ID, and 6% other neurodevelopmental conditions. Regarding occupation, 42% reported currently working, 35% studying, 12% being retired, 6% unemployed, and 4% sick leave. The remaining 1% described another occupation, such as being on parental leave. The general population group represented all 21 regions in Sweden, and their geographical representation reflected the general population in Sweden (see supplementary Table 1). Sample characteristics are detailed in Table 1.

**Table 1:** Sample characteristics

	All		Autistic		General population		Autistic vs. General population group
	Autistic group	General population group	< 15 years	15 and older	< 15 years	15 and older	
n	100	539	28	72	88	451	
Gender, female / male / other	55 / 41 / 4	327 / 206 / 6	10 / 16 / 2	45 / 25 / 2	54 / 30 / 4	273 / 176 / 2	*χ2* = 5.11, *p* = .078
Age, *M* (*SD*)	28.28 (14.85)	34.58 (18.69)	11.50 (1.35)	34.81 (12.35)	12.09 (1.45)	38.97 (17.29)	*t* = 3.73, *p* < .001
AQ-10, *M* (*SD*)	6.61 (2.13)	3.79 (2.08)	6.71 (1.98)	6.57 (2.19)	4.35 (2.17)	3.68 (2.05)	*t* = -12.22, *p* < .001
CAT-Q total score, *M* (*SD*)	98.51 (29.67)	81.87 (28.26)	82.71 (29.33)	104.65 (27.64)	80.23 (26.53)	82.19 (28.60)	*t* = -5.19, *p* < .001
CAT-Q Compensation, *M* (*SD*)	31.60 (12.97)	26.57 (13.13)	26.36 (14.80)	33.64 (11.68)	26.63 (12.03)	26.56 (13.35)	*t* = -3.55, *p* < .001
CAT-Q Masking, *M* (*SD*)	32.37 (11.41)	29.57 (8.79)	26.25 (10.61)	34.75 (10.88)	27.72 (8.07)	29.93 (8.88)	*t* = -2.33, *p* = .022
CAT-Q Assimilation, *M* (*SD*)	34.54 (10.83)	25.73 (9.93)	30.11 (9.38)	36.26 (10.92)	25.89 (9.62)	25.69 (10.00)	*t* = -7.57, *p* < .001

*Note.* Group differences were tested using Welch *t*-tests, except for the categorical variable gender where a Chi-squared test was used

Autistic participants were recruited via established contacts with Swedish interest organizations for autism and neurodevelopmental conditions (Autism Sweden, Society Attention, Organized Aspergers) and completed a web survey (hosted by Sunet Survey) comprising the questionnaires described below. A geographically and demographically representative general population sample was recruited supported by a marketing company (PFM Research) having access to large and well characterized Swedish panels. General population participants responded to the same questions, embedded into a web survey hosted by the company. A proportion of the participants recruited by the data collection company self-reported having an autism diagnosis (*n* = 49) and were therefore included in the autism sample. Autism diagnosis was self-reported and not clinically corroborated. Yet, autism diagnostic assessments in Swedish healthcare are typically conducted in accordance with national and regional guidelines (e.g., Ginsberg & Rahm, 2014), conducted by a multi-professional team (commonly including at least a medical doctor and a psychologist), and based on diagnostic criteria in ICD-10 or DSM-IV / DSM-5.

### Measures

#### CAT-Q

The CAT-Q is a self-report questionnaire developed based on descriptions of camouflaging behaviors by autistic adults, which was constructed to be appropriate for use in both autistic and non-autistic populations (Hull et al., 2018). The instrument comprises 25 items which are responded to on a seven-point Likert scale ranging from strongly disagree to strongly agree. Each item is scored from one to seven, where five items are reverse worded and thus reverse scored, yielding a total score of 25 to 175 with higher scores indicating more camouflaging behavior. In the original validation study, results from exploratory factor analysis and confirmatory factor analysis suggested a three-factor structure: Compensation (strategies used to compensate for challenges in social interaction and communication), Masking (strategies employed to present as less autistic), and Assimilation (strategies used to fit in with others in social situations) (Hull et al., 2018). The scale has demonstrated good internal consistency (*α* = 0.94) and test-retest stability (*r* = .77) as well as associations to theoretically linked constructs, including measures of autistic traits, depression, and generalized anxiety (*r* = .28 to 0.67).

#### CAT-Q/SE

The CAT-Q was translated from English to Swedish by authors KLR and SB. Difficulties arising in the translation process were resolved through correspondence with the authors of the original questionnaire. After the initial translation to Swedish, the questionnaire was back-translated to English by a professional and independent translator and subsequently authorized after minor adjustments. The Swedish version was then shared with experienced clinicians who piloted the questionnaire in clinical settings. After collecting feedback, adjustments were made where the verbal anchors were kept only for the extreme values (i.e., 1 and 7), as the piloting revealed that the other verbal descriptions were experienced as more confusing than helpful in the Swedish version.

#### Other Measures

All instruments used in the current study are validated and widely used self-report questionnaires. Autistic traits were measured using the Autism Spectrum Quotient-10 (AQ-10), which was developed from the original 50-item version of the AQ. In the original AQ-10 validation, the scale displayed high internal consistency (*α* = 0.85) and differentiated between autistic people and people without neurodevelopmental conditions (Allison et al., 2012). Recently, concerns have been raised regarding the internal consistency and factor structure of the AQ-10 (Jia et al., 2019; Taylor et al., 2020). However, a psychometric study of the Swedish version supported a unidimensional structure and found the internal consistency to be in an acceptable range (*α* = 0.67 – 0.73) for a such a brief scale measuring a heterogeneous construct (Lundin et al., 2019).

Symptoms of depression were measured with the Patient Health Questionnaire scale (PHQ-9) (Kroenke et al., 2001), which has been shown to display good psychometric properties, including good internal consistency (*α* = 0.86 – 0.89) and diagnostic validity. The instrument has been validated in autistic adolescents and adults (Arnold et al., 2020).

Anxiety was measured using the Generalized Anxiety Disorder scale (GAD-7; Spitzer et al., 2006), which has been validated in adolescent and adult populations, displaying good psychometric properties (Löwe et al., 2008). The GAD-7 is strongly correlated with anxiety measures including the Beck Anxiety Inventory (Spitzer et al., 2006) and provides a valid measure also of other anxiety disorders, e.g. social anxiety and panic disorder (Kroenke et al., 2007).

Quality of life was measured using the EUROHIS-QOL 8-item index, which is derived from the World Health Organization (WHO) Quality of Life Brief Scale (WHOQOL-BREF) (Power et al., 2003). The scale has shown sufficient reliability and validity across a multitude of countries (da Rocha et al., 2012; Schmidt et al., 2006).

Perceived stress was measured using the Stress in Children (SiC) questionnaire (Osika et al., 2007), for participants younger than 15 years. The SiC has shown good internal consistency (*α* = 0.86) and concurrent validity in children aged between 9 and 12 years. For participants who were 15 years and older the Perceived Stress Scale (PSS-14) was used, which displays adequate reliability including internal consistency (*α* = 0.84–0.86), as well as concurrent validity in adolescents and adults (Cohen et al., 1983).

As a crude measure of social functioning, the current study used the *Getting along* subscale from the WHO Disability Assessment Schedule 2.0 (WHODAS 2.0 for participants aged 15 or older and WHODAS Child for participants younger than 15 years) (Canino et al., 2013; Scorza et al., 2013; Üstün et al., 2010). The WHODAS is derived from the WHO International Classification of Functioning, Disability and Health (ICF) framework. Both the child and adult versions of the WHODAS display sufficient internal consistency, test-retest reliability, and concurrent validity (Scorza et al., 2013; Üstün et al., 2010).

### Procedure

The study was approved by the National Swedish Ethical Review Authority and all participants completed informed consent before accessing the questionnaires. Parent consent was required for participants under the age of 15, and these participants were recommended to have a parent close if they needed help with understanding questions but were instructed to respond according to their own lived experience. All participants were asked whether they agreed to be contacted again with the option to complete the CAT-Q/SE a second time, for test-retest analysis. Participants received a digital gift card (value of SEK 150, approximately $14) as compensation for participating. To avoid selection bias, the term camouflaging was not used in any recruitment adverts or study information. The study was conducted between June 2020 and October 2020.

### Analyses

Psychometric analyses of the CAT-Q/SE were computed following the principles of classical test theory. Analyses were conducted using R software, and *p*-values < 0.05 were considered significant.

*Item analyses* were conducted in the autism and general population samples separately. Item means and SDs were calculated to evaluate item difficulties. Item difficulty was standardized by summing all participant scores on an item and dividing with the sum obtained if all participants would have received the item max score (indicating the highest level of camouflaging, i.e. seven). Thus, standardized item difficulty values range between 0 and 1, where higher values indicate that more individuals display the behavior assessed by the specific item. Part-in-whole corrected item-total correlations describing the associations between single item scores and the total score of the CAT-Q/SE were conducted. Item validity, evaluating the power of each item to differentiate between autistic and non-autistic samples was examined by comparing the autism and general population groups using the Wilcoxon rank sum test.

*Reliability* was determined for the whole CAT-Q/SE scale and its subscales using the concepts of internal consistency in the total sample, and test-retest reliability (stability) in a subsample of 134 participants who completed the CAT-Q/SE a second time after an interval of 4 to 10 weeks after initial testing. Stability was calculated using Pearson correlation as well as a two-way mixed effects Intraclass Correlation Coefficients (ICC). ICC values within the range of 0.50 to 0.75 were interpreted as moderate, 0.75 to 0.90 as good, and > 0.90 as excellent reliability (Koo & Li, 2016). To determine the reliability of the questionnaire among individuals younger than 15 years (n = 116), internal consistency and test-retest stability (based on 58 participants) were also assessed separately in this group.

*Validity* was examined in terms of construct validity including diagnostic validity, concurrent validity, and factorial validity. Diagnostic validity was computed to assess the power of the CAT-Q/SE to differentiate between the autistic and general population samples, and between autistic females and autistic males, as hypothesized. Analyses of variance (ANOVA) was conducted in the full sample, testing the effect of group (autism or general population), gender, and the interaction effect autism by gender. An interaction effect in the full sample was followed up, testing the effect of gender on CAT-Q/SE total score in the autism and general population groups separately using *t*-tests, with Bonferroni corrected *p*-value threshold (two separate comparisons, i.e., *p*-values below 0.05 / 2 = 0.025 were considered significant). Only female and male gender were included in the analyses, as participants reported identifying as other gender, e.g., non-binary, were few (four in the autism group and six in the general population group). Means and SDs for females, males, and participants identifying as other gender are reported. In addition, receiver operating characteristic (ROC) curves were calculated to evaluate how accurately the total score classified participants as autistic or non-autistic, using the pROC package (Robin et al., 2011). ROC-analyses were conducted in the full sample as well as separately in participants younger than 15 years and in participants 15 years or older. Area under the curve (AUC) was calculated, describing the probability that the total score can distinguish between an autistic and a non-autistic individual completing the questionnaire (Mandrekar, 2010). AUC-values of 0.50 suggest no discrimination, 0.70 to 0.80 acceptable discrimination, 0.80 to 0.90 excellent, and ≥ 0.90 outstanding diagnostic validity (Hosmer & Lemeshow, 2000). The Youden index was used to determine a cut-off score yielding the combination of maximum specificity and sensitivity (Youden, 1950).

Concurrent validity was evaluated by assessing if the pattern of observed associations between the CAT-Q/SE scores and theoretically linked constructs matched the expected associations (Furr, 2011). Table 2 shows the hypothesized pattern of associations, presented as a hierarchy with the largest expected association first and the smallest last. We expected associations with self-reported autistic traits and social functioning to be stronger than associations with anxiety, depression, perceived stress, and quality of life, as the reverse could suggest confounding with other constructs such as social anxiety, as has been cautioned (Fombonne, 2020).

**Table 2:** Hypothesized pattern of associations between camouflaging and theoretically linked constructs for concurrent validity analysis

Construct	Expected strength of the association	Theoretical relationship
Autistic traits (AQ-10)	Moderate-to-large positive association	Autistic traits are linked to the core of the construct, camouflaging is suggested to encompass strategies to make existing elevated autistic traits less apparent (Hull et al., 2019).
Social functioning (Getting along subscale; WHODAS 2.0/WHODAS Child)	Moderate positive association (as higher WHODAS scores indicate more disability)	Lower social skills are believed to increase likelihood to use camouflaging (Hull et al., 2018), and such strategies might contribute to not getting the support needed, further decreasing social functioning (Livingston, Shah, et al., 2019)
Depressive symptoms (PHQ-9) Anxiety symptoms (GAD-7) Perceived stress (PSS-14/SiC) Quality of Life (EUROHIS-QOL)	Small-to-moderate positive associations, except quality of life where a negative association is expected	Theorized as detrimental consequences of camouflaging, e.g. exhaustion, experiences of presenting a “false” self, anxiety concerning whether camouflaging was “successful”, and reduced access to support (Bargiela et al., 2016; Cassidy et al., 2018; Hull et al., 2017, 2021; Lai et al., 2017).

Confirmatory factor analysis (CFA) was conducted to evaluate the fit of the factor structure reported for the original English CAT-Q (Hull et al., 2018), using the lavaan package (Rosseel, 2012). A good fit would indicate that the latent dimensions suggested for the original questionnaire are valid also for the Swedish version. The CFA fit indices were assessed using general cut-off values where good fit is indicated by Comparative Fit Index (CFI) ≥ 0.95, Root Mean Square Error of Approximation (RMSEA) ≤ 0.06, and Standardized Root Mean Square Residual (SRMR) ≤ 0.08 (Hu & Bentler, 1999). Diagonally Weighted Least Squares (DWLS) estimators were used to account for the ordinal Likert-scale data (DiStefano & Morgan, 2014), and robust statistics are reported. Exploratory factor analysis (EFA) was planned in case the previously identified factor structure would not fit the CAT-Q/SE data sufficiently, using Principal Axis Factoring with oblique rotation (promax). Factor extraction was determined based on a combination of the scree and the Kaiser criterion (i.e. retaining factors with eigenvalues > 1.0), and if needed by performing multiple EFAs with different factor structures to reach an parsimonious, balanced factor solution (Costello & Osborne, 2005). Factor loadings of 0.32 or higher were considered sufficient and items loading ≥ 0.32 on multiple factors were identified as cross-loading.

#### Effects of Age and Diagnostic Age, and CAT-Q/SE Norms

In order to make CAT-Q/SE total score distributions across age groups more accessible, scatter plots displaying the relation between age and total scores for the autistic and general population groups were fitted with locally estimated scatterplot smoothing (LOESS) curves. LOESS curves fit a regression line for data point x based on weighted least squares of data points in the neighborhood, meaning that the closest data points are given the largest weight. The LOESS curves are displayed with 95% confidence intervals. In addition, the association between age of autism diagnosis and CAT-Q/SE total score was assessed in the autism group.

To facilitate interpretation of CAT-Q/SE total scores, continuous norms for the general population and the autistic population were developed using polynomial regression models utilized by the cNORM package (Gary et al., 2021). In the general population group, norms were developed for age groups 10 to 15, 15 to 20, 20 to 40, 40 to 60, and 60 or older. For the autistic population the full autism group was used, as division into age groups would have resulted in too small subsets.

## Results

### Item Analyses

Part-in-whole corrected item-total correlations, item means and *SD*s, difficulty, and validity are presented in Table 3. All item-total correlations were positive, and most were *r*_it_ ≥0.30 in both the autism and general population sample, with exception for items 3, 12, 16, 19, 22, and 24. Regarding item validity, 17 of the total 25 items significantly discriminated between the autistic and the general population samples. Item difficulty was in the range *d*_i_ = 0.40 to 0.69 in the autism group, and between *d*_i_ =0.36 and 0.56 in the general population group.

**Table 3:** Item-total correlations (*r*_i−t_), item means, *SD*s, and item difficulty (*d*_i_) in autistic and general population groups as well as item validity

	Autistic group (n = 100)				General population group (n = 539)					
Item	*r*_i−t_	*M*	*SD*	*d*_i_	*r*_i−t_	*M*	*SD*	*d*_i_	*W* ^a^	*p*-value
1	0.51	3.0	1.9	0.43	0.66	2.9	1.8	0.42	26,316	0.702
2	0.66	3.7	2.1	0.53	0.68	3.5	1.7	0.50	25,928	0.541
3	0.25	4.8	2.0	0.69	0.26	3.4	1.9	0.49	16,586	< 0.001
4	0.73	3.5	2.0	0.51	0.72	2.9	1.8	0.41	21,967	0.003
5	0.47	3.5	2.1	0.51	0.66	2.9	1.9	0.42	22,397	0.006
6	0.55	4.4	2.0	0.62	0.62	3.9	1.8	0.56	23,083	0.021
7	0.76	4.3	2.2	0.61	0.76	3.0	1.8	0.43	17,924	< 0.001
8	0.77	4.2	2.0	0.60	0.77	3.3	1.8	0.47	19,568	< 0.001
9	0.56	4.5	2.1	0.64	0.51	4.1	1.7	0.59	23,748	0.056
10	0.30	4.1	2.1	0.58	0.72	2.9	1.8	0.42	18,549	< 0.001
11	0.51	2.8	1.9	0.40	0.74	2.5	1.8	0.36	24,237	0.092
12	0.32	4.1	2.2	0.59	0.02	3.7	1.9	0.53	23,676	0.051
13	0.69	4.0	2.0	0.57	0.74	3.2	1.8	0.45	21,007	< 0.001
14	0.70	4.5	2.0	0.64	0.69	3.4	1.9	0.49	18,426	< 0.001
15	0.76	3.9	2.1	0.55	0.78	3.3	1.8	0.48	23,085	0.021
16	0.28	3.8	2.1	0.55	0.69	3.2	1.9	0.46	22,068	0.003
17	0.54	3.3	2.3	0.47	0.65	2.8	1.9	0.39	23,526	0.036
18	0.33	4.0	2.0	0.56	0.51	3.7	1.7	0.52	25,063	0.260
19	0.37	4.6	2.0	0.65	0.28	3.3	1.8	0.47	17,324	< 0.001
20	0.60	3.5	2.0	0.49	0.72	3.0	1.8	0.43	23,507	0.038
21	0.72	3.5	2.0	0.50	0.73	3.4	1.8	0.48	26,192	0.650
22	0.21	4.5	1.9	0.64	0.23	3.4	1.6	0.48	17,655	< 0.001
23	0.57	3.2	2.0	0.46	0.73	2.8	1.8	0.41	24,242	0.101
24	0.43	4.4	2.1	0.63	0.08	3.9	1.9	0.56	23,464	0.037
25	0.70	4.6	2.2	0.65	0.74	3.2	1.9	0.46	17,416	< 0.001

*Note.*^a^ All groupwise comparisons were conducted using Wilcoxon rank sum test, *r*_i−t_ = item-total correlation,

*d*_i_ = item difficulty

### Reliability

Internal consistency was excellent for the CAT-Q/SE total scale (all 25 items) in the full sample (Cronbach’s *α* = 0.93), and good-to-excellent for the subscales, Compensation (*α* = 0.92), Masking (*α* = 0.78), and Assimilation (*α* = 0.85). Consistency for the total scale was similar in the autistic (*α* = 0.92) and general population (*α* = 0.94) samples, whereas some descriptive variation was found in the Compensation (*α* = 0.88 vs. *α* = 0.93), Masking (*α* = 0.84 vs. *α* = 0.76), and Assimilation (*α* = 0.81 vs. *α* = 0.85) subscales.

The CAT-Q/SE displayed good test-retest reliability regarding the total score (*r* = .85; p < .001; *ICC* = 0.85, *p* < .001). Similar results were found in the separate subscales, Compensation (*r* = .82, *p* < .001; *ICC* = 0.82, *p* < .001), Masking (*r* = .78; *p* < .001; *ICC* = 0.78, *p* < .001), and Assimilation (*r* = .86, *p* < .001; *ICC* = 0.85, *p* < .001). Test-retest reliability was descriptively higher in the autistic group compared to the general population group, for both the total score (*r* = .90 vs. *r* = .79), and the subscales: Compensation (*r* = .89 vs. *r* = .78), Masking (*r* = .84 vs. *r =* .68), and Assimilation (*r* = .88 vs. *r* = .76), all *p*-values < 0.001.

Among the 116 participants younger than 15 years, the internal consistency for the CAT-Q/SE total scale was excellent (*α* = 0.92), and good-to-excellent for subscales Compensation (*α* = 0.92), Masking (*α* = 0.75), and Assimilation (*α* = 0.81). Test-retest reliability for the total score was moderate-to-good in this subsample (*r* = .80, *p* < .001, *ICC* = 0.79, *p* < .001), as well as for subscales Compensation (*r* = .79, *p* < .001; *ICC* = 0.79, *p* < .001), Masking (*r* = .73, *p* < .001; *ICC* = 0.73, *p* < .001), and Assimilation (*r* = .76, *p* < .001; *ICC* = 0.76, *p* < .001).

### Validity

Assessing diagnostic validity, the ANOVA showed a main effect of group (autism or general population) on CAT-Q/SE total scores (*F*(1,625) = 23.54, *p* < .001, partial *η*^*2*^ = 0.04), and an interaction effect between group and gender (*F*(1,625) = 8.79, *p* = .003. Follow-up analyses revealed that autistic females scored higher compared to autistic males (*M* = 105.2, *SD* = 29.9 vs. *M* = 90.2 *SD* = 28.1), corresponding to a medium effect (*t*(89.08) = 2.51, *p* = .014, *g* = 0.51), while no difference was found between females (*M* = 80.3, *SD* = 29.4) and males (*M* = 84.2, *SD* = 25.9) in the general population (*t*(475.92) = -1.61, *p* = .109). Few participants identified as other gender than female or male in the autism group (*n* = 4; CAT-Q/SE total score: *M* = 91.5, *SD* = 25.1), and in the general population group (*n* = 6; CAT-Q/SE total score: *M* = 83.5, *SD* = 38.2).

**Fig. 1:**
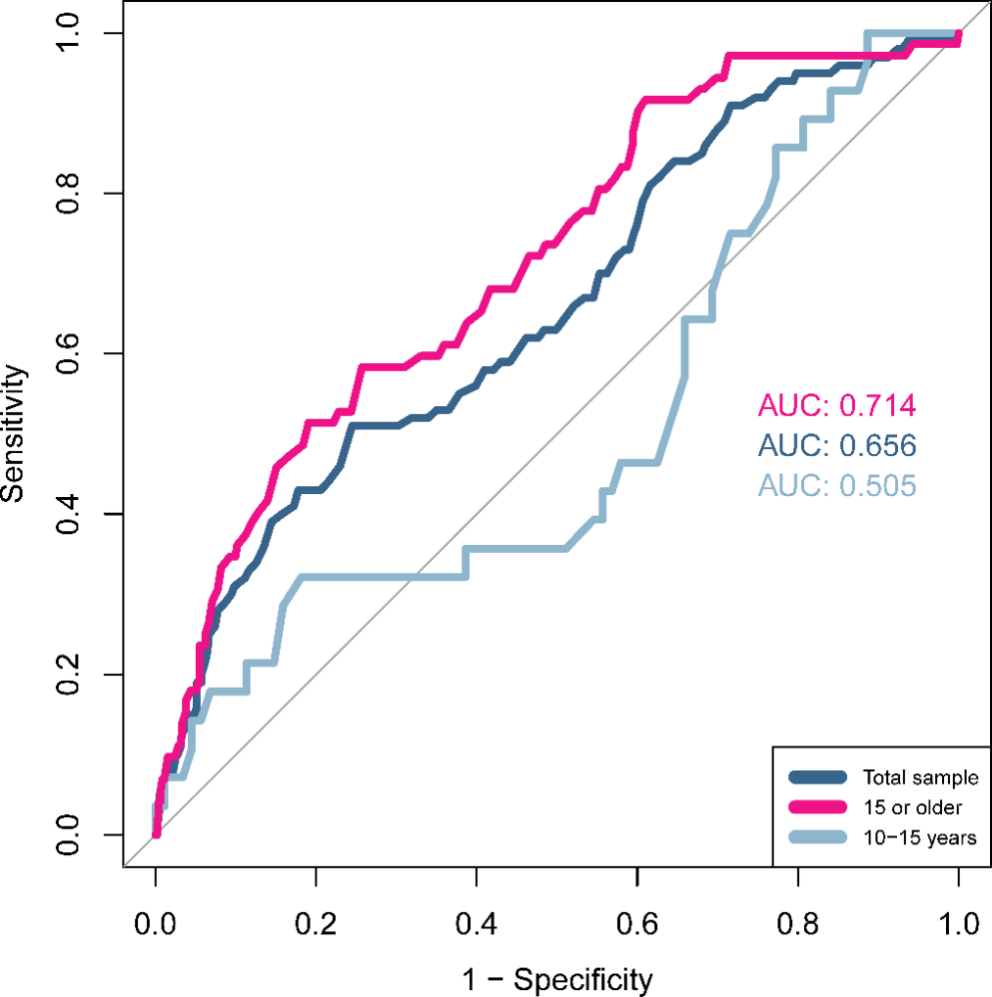
Receiver Operating Characteristics curves of the CAT-Q/SE total score for the full sample as well as separately for participants 15 years or older and children younger than 15

The ROC-curve for the total sample, as well as separate curves for participants younger than 15 years and participants 15 years or older are shown in Fig. 1. The CAT-Q/SE total score did not discriminate particularly well between autistic and non-autistic participants in the full sample, as indicated by the *AUC* being below the acceptable cut-off (*AUC* = 0.656). For participants younger than 15 years the classification of the total score was also below acceptable (*AUC* = 0.505), whereas for participants 15 or older, the ROC-curve indicated acceptable diagnostic validity (*AUC* = 0.714). In the full sample, a total score of 39.5 gave the highest sensitivity (0.99), while 157 gave perfect specificity (1.00). A total score of 102.5 (i.e., cut-off at 103 or higher) yielded the maximum combination of sensitivity (0.51) and specificity (0.76). Among participants younger than 15 years, the 102.5 cut-off provided sensitivity and specificity values of 0.32 and 0.82 respectively, whereas the same score in participants 15 years or older resulted in sensitivity 0.58 and specificity 0.74.

In the full sample, concurrent correlations of the expected sizes were found between CAT-Q/SE scores and measures of theoretically linked constructs with few exceptions, see Table 4. Some associations did not fit the expected pattern, where social functioning difficulties (WHODAS 2.0; *r* = .52), depression (PHQ-9; *r* = .48), anxiety (GAD-7; *r* = .45), and perceived stress (PSS-14; *r* = .42) showed similar or stronger associations to CAT-Q/SE total scores compared to autistic traits measured with the AQ-10 (*r =* .44). See Table 5 for the associations in the subsample younger than 15 years, which largely reflected the pattern found in the older subsample. However, in this age group, the association between CAT-Q/SE total score and autistic traits was weaker (*r* = .28). Also, no associations were found between the Masking and Compensation scores and autistic traits or quality of life, and neither between the Compensation subscale and perceived stress.

**Table 4:** Concurrent validity of the CAT-Q/SE (total and subscale scores) with theoretically linked measures

	AQ-10	WHODAS 2.0 Getting along subscale^a^	PHQ-9	GAD-7	PSS-14^a^	EUROHIS-QOL
CAT-Q/SE total score	0.44 (*p* < .001)	0.52 (*p* < .001)	0.48 (*p* < .001)	0.45 (*p* < .001)	0.42 (*p* < .001)	− 0.23 (*p* < .001)
CAT-Q/SE Compensation	0.38 (*p* < .001)	0.47 (*p* < .001)	0.43 (*p* < .001)	0.39 (*p* < .001)	0.29 (*p* < .001)	− 0.09 (*p* = .031)
CAT-Q/SE Masking	0.25 (*p* < .001)	0.35 (*p* < .001)	0.31 (*p* < .001)	0.31 (*p* < .001)	0.29 (*p* < .001)	− 0.13 (*p* = .001)
CAT-Q/SE Assimilation	0.53 (*p* < .001)	0.53 (*p* < .001)	0.50 (*p* < .001)	0.47 (*p* < .001)	0.55 (*p* < .001)	− 0.42 (*p* < .001)

*Note*. ^a^ Calculated only for participants 15 years or older (n = 523)

Autistic traits (AQ-10), social functioning (WHODAS 2.0, Getting along subscale), depression (PHQ-9), anxiety (GAD-7), perceived stress (PSS-14), and quality of life (EUROHIS-QOL).

**Table 5:** Concurrent validity of the CAT-Q/SE with theoretically linked measures in individuals aged 10–15 years^a^

	AQ-10	WHODAS Child Getting along subscale	PHQ-9	GAD-7	SiC	EUROHIS-QOL
CAT-Q/SE total score	0.28 (p = .002)	0.44 (*p* < .001)	0.48 (*p* < .001)	0.41 (*p* < .001)	0.30 (*p* = .001)	− 0.26 (*p* = .005)
CAT-Q/SE Compensation	0.17 (*p* = .070)	0.33 (*p* < .001)	0.43 (*p* < .001)	0.35 (*p* < .001)	0.16 (*p* = .084)	− 0.08 (*p* = .378)
CAT-Q/SE Masking	0.09 (*p* = .326)	0.27 (*p* = .003)	0.36 (*p* < .001)	0.32 (*p* < .001)	0.18 (*p* = .048)	− 0.13 (*p* = .174)
CAT-Q/SE Assimilation	0.48 (*p* < .001)	0.56 (*p* < .001)	0.46 (*p* < .001)	0.42 (*p* < .001)	0.46 (*p* < .001)	− 0.50 (*p* < .001)

*Note.*^a^ n = 116,

Autistic traits (AQ-10), social functioning (WHODAS Child, Getting along subscale), depression (PHQ-9), anxiety (GAD-7), perceived stress (SiC), and quality of life (EUROHIS-QOL).

Regarding factorial validity, the model fit for the original three factor structure was insufficient, as indicated by fit indices values not being within the acceptable ranges (*CFI* = 0.828, *RMSEA* (90% *CI*) = 0.157 (0.153–0.161), *SRMR* = 0.091). Therefore, an EFA was conducted, which supported a two-factor solution accounting for 51% of the variance in the sample, where factor 1 (eigenvalue 10.19) comprised all items except the five reverse coded items, which loaded to factor 2 (eigenvalue 2.46). All factor loadings were > 0.32, with no cross-loadings. The correlation between the two factors was 0.22. See Supplementary Table 2 for factor loadings of the 25 items. To evaluate if removing the reversed items would improve model fit for the original factor structure, a follow-up CFA with the remaining 20 items was conducted, which showed improved model fit (*CFI* = 0.961, *RMSEA* (90% *CI*) = 0.091 (0.085–0.096), *SRMR* = 0.040). The internal consistency for the remaining 20 items was excellent for the total scale (*α* = 0.95), and good-to-excellent in the Compensation (*α* = 0.92), Masking (*α* = 0.88), and Assimilation subscales (*α* = 0.88).

### Effects of Age and Diagnostic Age, and CAT-Q/SE Norms

**Fig. 2:**
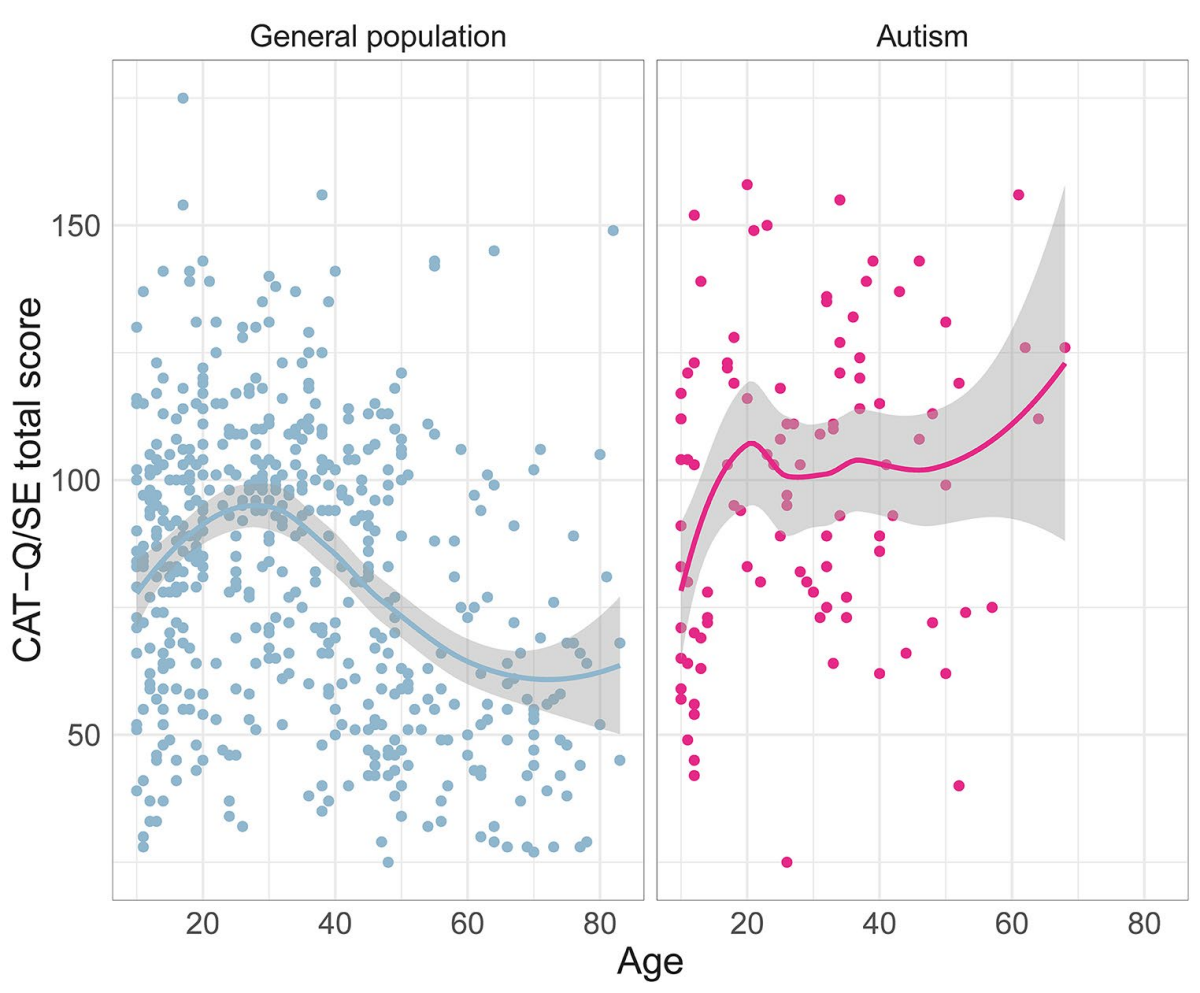
Scatter plots and LOESS regression curves with 95% confidence intervals visualizing age and CAT-Q/SE total score for the general population and autism groups

See Fig. 2 for scatter plots of the relation between age and CAT-Q/SE total score among autistic and general population participants. In the general population, total scores increased from childhood to adulthood reaching a plateau in young adulthood (ages 20 to 40) and steadily decreasing in older age groups. Among the autistic participants, a corresponding increase is seen in adolescence, but in contrast to the non-autistic group the LOESS curve does not suggest a clear reduction of camouflaging during adulthood. Regarding age of diagnosis among the autistic participants, the age when diagnosed with autism correlated moderately with CAT-Q/SE total score (*r* = 0.30, *p* = .003), suggesting that camouflaging was higher among those diagnosed later in life. CAT-Q/SE total score norms including raw scores, T-scores, and percentiles for the general population, divided in age groups 10 to 15, 15 to 20, 20 to 40, 40 to 60, and 60 or older, as well as for the autistic group are presented in Supplementary Tables 3–8.

## Discussion

This study describes the psychometric evaluation of the Swedish version of the CAT-Q in samples of autistic and non-autistic people. The scale displayed good-to-excellent reliability in the full sample, and similar estimates among participants aged 10 to 15 years. Construct validity of the CAT-Q/SE was supported by autistic participants scoring higher than the general population sample, autistic females scoring higher than autistic males, and by associations between CAT-Q/SE scores and theoretically neighboring constructs. However, some associations did not follow the expected pattern, and the factor structure reported for the original CAT-Q was not supported in the Swedish data set for the 25 item scale, raising potential issues regarding construct validity. Furthermore, different age trends were observed in the samples, suggesting that these behaviors follow separate trajectories in autistic and non-autistic people.

The corrected item-total correlations were in general acceptable, but six items failed to display correlations ≥ 0.30 in both the autistic and general population samples. Five of these were reversed items suggesting that these are not as consistent with the scale as a whole compared to the other items. (The reversed items are further discussed in relation to the factor structure below.) Item difficulties for all 25 items were within the preferred range of 0.20 to 0.80, suggesting that the items display adequate difficulty (not being endorsed by too few or too many participants) supporting their discriminatory capability. While the majority (17 of 25) of the CAT-Q/SE items differentiated between the autistic and the general population samples as expected, the content of the items that did not could provide clues regarding less autism-specific aspects of camouflaging. For example, autistic and non-autistic participants did not differ on items 2, 18, and 21, which cover attempts to appear relaxed in social interaction and being constantly aware of the impression made on others. Similar behaviors have been described in connection to camouflaging by autistic adults (Hull et al., 2017), but these items could also tap experiences that are common among non-autistic people and especially people suffering from social anxiety, posing a risk that these items measure other constructs than the intended. On the other hand, items that differentiated between the groups might cover more autism-specific aspects of camouflaging, including experiences of “performing” rather than being oneself in social interaction (item 7), attempting to improve understanding of social skills by watching others (item 14), and learning how others use non-verbal communication by watching television or reading fiction (item 20).

Regarding reliability, the CAT-Q/SE demonstrated good-to-excellent internal consistency and stability in the full sample, and good-to-excellent internal consistency and generally good test-retest reliability in the subsample of participants aged between 10 and 15 years. Our data thus indicate that the scale displays low levels of measurement error also when completed by children, supporting that it is a reliable measure for people with a minimum age of 10 years. It should be noted that the youngest participants were instructed to have a parent close for potential questions and the reliability reported for this age group should therefore be considered as applicable to children completing the questionnaire with available support nearby. Furthermore, research focusing on camouflaging in autistic people with concurrent ID is scarce, and while the current study included eight participants self-reporting ID (five autistic and three in the general population sample), the reliability of the CAT-Q/SE in this population is currently unknown.

We observed inconsistent findings for indicators of construct validity. Supporting the validity of the CAT-Q/SE, the autistic group displayed higher total scores than the general population group as hypothesized. However, the ROC-analyses indicated acceptable precision in differentiating between autistic and non-autistic people only among those aged 15 years or more. Also, the sensitivity and specificity of the 102.5 cut-off were modest even in this group (0.58 and 0.74), indicating that the cut-off should not be used to make diagnostic judgements in autism assessments. Importantly, the CAT-Q was developed as a measure of camouflaging and not an autism diagnostic instrument or a measurement of autistic traits, and while camouflaging experiences are likely common among autistic people, not everyone on the autism spectrum camouflages extensively (Cage & Troxell-Whitman, 2019; Hull et al., 2017). Therefore, lower sensitivity and specificity values than what is reported for diagnostic instruments is to be expected from the CAT-Q/SE. Overall, the difference between the autistic and general population groups was smaller than what was reported among adults in the original validation study (Hull et al., 2018), which should be interpreted in light of the current study also including younger age groups, who appear to self-report lower levels of camouflaging. Also, in the current study 49% of the autistic participants were diagnosed before the age of 18 years, whereas the majority of autistic participants in the English validation study were diagnosed late in life (only 12% in childhood), which might also be reflected in camouflaging scores as indicated by age of diagnosis showing a positive association with camouflaging in our data. The lack of differentiation in participants younger than 15 years could mean that the ability of the CAT-Q/SE to pick up camouflaging behaviors is not as good in autistic children compared to older age groups. The lower scores among autistic children in our study could also be influenced by the lower proportion of girls in this group. Still, this finding might also suggest that autistic children camouflage to a lesser extent compared to autistic adolescents and adults, i.e., that there is no true group-level difference between autistic and non-autistic children. Support for this is suggested by a recent study were young autistic participants (aged 13 to 19 years) self-reported lower CAT-Q total scores (*M* = 93.3) than what has been found among autistic adults (Jedrzejewska & Dewey, 2022).

In accordance with our hypothesis, autistic females received higher CAT-Q/SE total scores than autistic males. Sex/gender differences in camouflaging have not been found in all previous studies (Cook et al., 2021), but have been suggested by a range of studies, including qualitative accounts from professionals working with autistic people (Lundin et al., 2021), studies using self-report (Cassidy et al., 2018; Hull et al., 2019) and discrepancy approaches (Cola et al., 2020; Lai et al., 2017; Schuck et al., 2019). Our results add to the previous data suggesting that autistic females are especially prone to camouflaging, in contrast to non-autistic females who do not appear to use similar behaviors more than males. Nevertheless, it should be acknowledged that camouflaging is reported also among autistic males and should not be perceived as behaviors found exclusively among autistic females (Cook et al., 2021).

The concurrent validity of the CAT-Q/SE was partially supported by our data, where scores were associated with constructs that are theoretically linked to camouflaging. However, associations with mental health problems, e.g., anxiety and depression, stood out as being stronger than hypothesized. While these associations might reflect detrimental consequences of camouflaging, correlations with psychiatric symptoms were often similar to or stronger than those found with autistic traits, suggesting a risk of confounding by psychopathology. Similarly, the association between the CAT-Q/SE and social functioning challenges measured by the WHODAS 2.0/Child was stronger than the association found with autistic traits, implying that camouflaging might be a response to challenges in social interaction in general rather than to specific autistic characteristics. An alternative interpretation is that the AQ-10 items cover behaviors such as sensory hypersensitivity, special interests, and challenges with simultaneous capacity, that might not be as closely related to the camouflaging operationalization in the CAT-Q as the WHODAS-items are (which also likely overlap substantially with autistic traits in the social domain). Still, the above could suggest that the CAT-Q/SE picks up on behaviors that are not specifically linked to autistic camouflaging, but generally with atypical traits and behavioral challenges, as recently discussed (Fombonne, 2020; Williams, 2022).

The associations between camouflaging and psychiatric symptoms, perceived stress, and reduced quality of life, found both in the full sample and among those younger than 15 years, require further investigation, including elucidation of whether the link is causal, i.e. whether the use of camouflaging behaviors contribute to detrimental outcomes as has been suggested (Bargiela et al., 2016; Hull et al., 2021; Lai et al., 2017), or whether common confounders influence both camouflaging and detrimental outcomes. Potential confounders could be factors that has shown associations with camouflaging as well as with mental health problems or wellbeing, such as autism-related stigma (Perry et al., 2022) or the personality factor neuroticism (Robinson et al., 2020). Further research in this area could provide valuable information regarding whether camouflaging is a fruitful target for interventions attempting to alleviate mental health problems in autism.

The original three factor solution found by Hull and colleagues (2018) did not fit the CAT-Q/SE data sufficiently. There could be several reasons for this, including cultural differences in social behavior or that the Swedish translation added slightly different meanings to items, despite the process of back-translation and validation from the first author of the English version. Results from EFA instead suggested a two-factor solution, where reversed items loaded to a separate factor. This partly reflects findings from the Japanese CAT-Q validation, where an exploratory factor analysis in the non-autistic subsample suggested a four-factor structure, where only reversed items loaded to a fourth factor (Hongo, 2022). The two-factor structure suggested by our EFA does not clearly match the theoretical model of camouflaging, but rather suggest that participants may have responded in a specific way to the reversed questions. Reverse-worded items can be more difficult to comprehend for respondents, thus increasing the risk of confounding by constructs that the scale is not intended to measure (e.g., verbal ability) (Suárez Álvarez et al., 2018). A 20-item version of the CAT-Q/SE with the reverse-worded items removed, improved the model fit for the original three factor solution and displayed promising reliability, suggesting that it might be worthwhile to further test if construct validity is improved if reversed items are removed or presented in positively worded form.

### Age Effects on Camouflaging

The trend curve in the general population sample suggested an increase in camouflaging during adolescence and young adulthood followed by a gradual decrease in adulthood, whereas in the autistic sample camouflaging levels remained elevated during adulthood. Consistent with the increase of camouflaging behaviors among non-autistic adolescents seen in our data, a recent study in neurotypical adolescents found that camouflaging scores were higher in ages 16 to 18 years than in 13 to 15 years, potentially explained by increasing conformity pressure, and greater awareness and concern of the impression made on others in this age (Jorgenson et al., 2020). Given that camouflaging shows an association with neuroticism (Robinson et al., 2020), the decrease seen during adulthood in the general population group could be influenced by the general increase in the emotional stability personality trait (the reverse of neuroticism) during adulthood, and experiences of being more settled in professional and private life (Roberts et al., 2006). Such experiences could contribute to non-autistic adults being less concerned about others’ impressions of themselves. In contrast, autistic adults might not experience a reduction of external demands described as motivators for camouflaging, e.g., that it is required to be accepted in society (Cage & Troxell-Whitman, 2019; Hull et al., 2017). Marginalized groups, here autistic people, might be prone to use camouflaging (or impression management) as strategies to mitigate risks or reach a “baseline” of social favorableness, whereas non-marginalized groups use such behaviors more voluntarily to increase social favorability and “appear better than others” (Ai et al., 2022). If camouflaging persists at an elevated level in autistic adults as suggested by our data, it might a pose a risk factor for detrimental outcomes throughout adulthood. Still, recent evidence suggests that anxiety is reduced in older autistic adults (50 to 71 years) compared to younger (19 to 48 years) (Yarar et al., 2022), suggesting that the effect observed in the general population may arrive in autism, yet later in life.

### Limitations

This study had some limitations that deserve to be addressed. First, autism diagnosis was self-reported, not clinically corroborated. However, a substantial proportion of the autistic participants were recruited through interest organizations for autism and neurodevelopmental conditions, and previous research indicates that diagnosis reported in web-based studies are valid. For example, the vast majority of parent-reported autism diagnosis can be confirmed by medical records (Daniels et al., 2012). In addition, the autism sample scored significantly higher on the AQ-10 than the general population group. Second, all measures used in the current study were self-reported which might be a source of bias. On balance, key constructs in the study, such as camouflaging, quality of life, perceived stress, and anxiety, are predominantly defined by primary perspective experiences, making self-report an appropriate method of measurement. In addition, the web survey format allowed us to collect data from a reasonably large sample. Third, while the self-report measures used in the current study are validated in adolescents and adults, several of them have not been sufficiently validated in children. However, we observed comparable associations with the CAT-Q/SE scores among the adults and children in our sample. In addition, autistic children and adolescents have been reported to provide accurate self-reports, including measures of emotional and behavioral problems (Bakhtiari et al., 2021). Fourth, the use of the AQ-10 as the sole measure of autistic traits in the current study is a potential methodological limitation. The psychometric properties of the scale have been questioned, and while a large Swedish study reported adequate validity, the reliability was not more than acceptable (Lundin et al., 2019). Fifth, the sample of autistic participants younger than 15 years was relatively small (n = 28), limiting the generalizability of our findings in this age group where collected CAT-Q/SE scores might not fully reflect the variation in the population of autistic children. Sixth, we sought a higher representation of females than males in the current study to ensure that the sample included a sufficient proportion of autistic females, who appear to use camouflaging more extensively (Cook et al., 2021), and thus also form the main target group of the CAT-Q/SE in clinical settings. However, the gender representation differed between age groups, where autistic participants younger than 15 years included proportionally more boys, whereas the group of autistic participants aged 15 years or older included more females than males. This could have led to that the acquired CAT-Q/SE scores in the group of autistic participants younger than 15 years underestimate the true score in this population, if gender differences in camouflaging are already present at this age which is suggested by previous research (Cook et al., 2021). Seventh, the sample included eight participants self-reporting an ID. These participants did not self-report notably low or high scores on the CAT-Q/SE and were therefore not excluded from the analyses. Eight, the autistic sample was slightly younger than the general population sample, which might entail that group comparisons somewhat underestimate the true difference in CAT-Q/SE scores. Ninth, it should be noted that the trend curves displaying age effect on camouflaging are exploratory and cross-sectional in nature and require future validation, preferably using longitudinal designs.

## Conclusion

The Swedish version of the CAT-Q showed good-to-excellent reliability and some support for construct validity: we could particularly confirm the expected pattern with higher camouflaging scores in autistic individuals, and foremost in autistic females, as well as consistent correlations with neighboring constructs. On the other hand, diagnostic validity was limited, and the factor structure of the original CAT-Q was not confirmed. Construct validity issues were particularly observable in children aged between 10 and 15 years, and the CAT-Q/SE should thus be used only exploratory and with necessary clinical caution in this age group. Overall, our analyses support that the CAT-Q/SE could be a valuable addition to autism assessments and research in Swedish settings, yet further investigation of the construct validity of the scale is warranted including whether camouflaging is specifically related to autism or rather is related to a wider range of clinical traits. Also, the CAT-Q/SE construct validity might be improved by omitting reversed items or making all items positively worded. Finally, our data suggests differential age trajectories of camouflaging: in non-autistic people these behaviors appear to diminish during adulthood, while remaining at an elevated level during adulthood among autistic people.

### Electronic supplementary material

Supplementary Material 1
