# Supplementary material for: Camouflaging in Autism: Age Effects and Cross-Cultural Validation of the Camouflaging Autistic Traits Questionnaire (CAT-Q)
Source: J Autism Dev Disord. 2023 Feb 9;54(5):1749–64. doi: 10.1007/s10803-023-05909-8 (PMC11136743; doi:10.1007/s10803-023-05909-8)
Supplement: Supplementary file 1 — Supplementary Material 1 [file 10803_2023_5909_MOESM1_ESM.docx]

**Supplementary material**

**Supplementary Table 1.** Geographical representation of autism and general population group

|  | Autism group | | General population group | | Population of Sweden^a^ | |
| --- | --- | --- | --- | --- | --- | --- |
| Region | n | Proportion | n | Proportion | n | Proportion |
| Blekinge | 3 | 3% | 13 | 2.4% | 159 056 | 1.5% |
| Dalarna | 1 | 1% | 9 | 1.7% | 287 676 | 2.8% |
| Gotland | 2 | 2% | 8 | 1.5% | 60 124 | 0.6% |
| Gävleborg | 2 | 2% | 7 | 1.3% | 287 502 | 2.8% |
| Halland | 1 | 1% | 22 | 4.1% | 336 748 | 3.2% |
| Jämtland | 1 | 1% | 4 | 0.7% | 131 155 | 1.3% |
| Jönköping | 4 | 4% | 11 | 2.0% | 365 010 | 3.5% |
| Kalmar | 0 | 0% | 17 | 3.2% | 246 010 | 2.4% |
| Kronoberg | 2 | 2% | 14 | 2.6% | 202 263 | 1.9% |
| Norrbotten | 2 | 2% | 7 | 1.3% | 249 614 | 2.4% |
| Skåne | 11 | 11% | 72 | 13.4% | 1 389 336 | 13.4% |
| Stockholm | 26 | 26% | 130 | 24.1% | 2 391 990 | 23.0% |
| Södermanland | 3 | 3% | 20 | 3.7% | 299 401 | 2.9% |
| Uppsala | 4 | 4% | 17 | 3.2% | 388 394 | 3.7% |
| Värmland | 4 | 4% | 18 | 3.3% | 282 885 | 2.7% |
| Västerbotten | 1 | 1% | 15 | 2.8% | 273 192 | 2.6% |
| Västernorrland | 2 | 2% | 15 | 2.8% | 244 554 | 2.4% |
| Västmanland | 3 | 3% | 14 | 2.6% | 277 141 | 2.7% |
| Västra Götaland | 15 | 15% | 86 | 16.0% | 1 734 443 | 16.7% |
| Örebro | 8 | 8% | 16 | 3.0% | 305 643 | 2.9% |
| Östergötland | 5 | 5% | 24 | 4.5% | 467 158 | 4.5% |

*Note*. ^a^ Official statistics (SCB, 2020).

**References**

SCB. (2020). *Folkmängd i riket, län och kommuner 31 december 2020 och befolkningsförändringar 2020*. Statistiska Centralbyrån. https://www.scb.se/hitta-statistik/statistik-efter-amne/befolkning/befolkningens-sammansattning/befolkningsstatistik/pong/tabell-och-diagram/folkmangd-och-befolkningsforandringar---helarsstatistik/folkmangd-i-riket-lan-och-kommuner-31-december-2020-och-befolkningsforandringar-2020/

**Supplementary Table 2.** EFA factor loadings of the 25 items in CAT-Q/SE

| Item | Factor 1 | Factor 2 |
| --- | --- | --- |
| 1 | **.711** | -.118 |
| 2 | **.709** | -.035 |
| 4 | **.752** | .043 |
| 5 | **.683** | -.015 |
| 6 | **.684** | -.093 |
| 7 | **.701** | .266 |
| 8 | **.798** | .060 |
| 9 | **.558** | -.024 |
| 10 | **.619** | .190 |
| 11 | **.778** | -.089 |
| 13 | **.705** | .182 |
| 14 | **.719** | .074 |
| 15 | **.806** | .006 |
| 16 | **.563** | .248 |
| 17 | **.728** | -.118 |
| 18 | **.601** | -.220 |
| 20 | **.780** | -.087 |
| 21 | **.808** | -.122 |
| 23 | **.784** | -.095 |
| 25 | **.676** | .278 |
| 3 (rev) | .059 | **.611** |
| 12 (rev) | -.081 | **.390** |
| 19 (rev) | .036 | **.778** |
| 22 (rev) | -.024 | **.769** |
| 24 (rev) | -.074 | **.543** |

*Note*. Factor loadings ≥.32 are presented in bold.

**Supplementary Table 3.** CAT-Q/SE norms autism group

| CAT-Q/SE total score | T-score | Percentile | CAT-Q/SE total score | T-score | Percentile |
| --- | --- | --- | --- | --- | --- |
| <32 | 24.24 | 0.5 | 76 | 43 | 24.2 |
| 32 | 24.79 | 0.6 | 77 | 43.32 | 25.2 |
| 33 | 25.41 | 0.7 | 78 | 43.65 | 26.3 |
| 34 | 26 | 0.8 | 79 | 43.96 | 27.3 |
| 35 | 26.58 | 1 | 80 | 44.28 | 28.4 |
| 36 | 27.14 | 1.1 | 81 | 44.6 | 29.5 |
| 37 | 27.68 | 1.3 | 82 | 44.91 | 30.5 |
| 38 | 28.2 | 1.5 | 83 | 45.23 | 31.7 |
| 39 | 28.72 | 1.7 | 84 | 45.54 | 32.8 |
| 40 | 29.22 | 1.9 | 85 | 45.85 | 33.9 |
| 41 | 29.7 | 2.1 | 86 | 46.16 | 35.1 |
| 42 | 30.18 | 2.4 | 87 | 46.47 | 36.2 |
| 43 | 30.65 | 2.6 | 88 | 46.78 | 37.4 |
| 44 | 31.11 | 2.9 | 89 | 47.09 | 38.5 |
| 45 | 31.56 | 3.3 | 90 | 47.39 | 39.7 |
| 46 | 32 | 3.6 | 91 | 47.7 | 40.9 |
| 47 | 32.43 | 3.9 | 92 | 48 | 42.1 |
| 48 | 32.86 | 4.3 | 93 | 48.31 | 43.3 |
| 49 | 33.28 | 4.7 | 94 | 48.61 | 44.5 |
| 50 | 33.7 | 5.2 | 95 | 48.92 | 45.7 |
| 51 | 34.1 | 5.6 | 96 | 49.22 | 46.9 |
| 52 | 34.51 | 6.1 | 97 | 49.52 | 48.1 |
| 53 | 34.9 | 6.6 | 98 | 49.83 | 49.3 |
| 54 | 35.29 | 7.1 | 99 | 50.13 | 50.5 |
| 55 | 35.68 | 7.6 | 100 | 50.43 | 51.7 |
| 56 | 36.06 | 8.2 | 101 | 50.74 | 52.9 |
| 57 | 36.44 | 8.8 | 102 | 51.04 | 54.1 |
| 58 | 36.81 | 9.4 | 103 | 51.34 | 55.3 |
| 59 | 37.18 | 10 | 104 | 51.64 | 56.5 |
| 60 | 37.55 | 10.7 | 105 | 51.95 | 57.7 |
| 61 | 37.91 | 11.3 | 106 | 52.25 | 58.9 |
| 62 | 38.27 | 12 | 107 | 52.55 | 60.1 |
| 63 | 38.62 | 12.8 | 108 | 52.86 | 61.2 |
| 64 | 38.98 | 13.5 | 109 | 53.16 | 62.4 |
| 65 | 39.33 | 14.3 | 110 | 53.47 | 63.6 |
| 66 | 39.67 | 15.1 | 111 | 53.77 | 64.7 |
| 67 | 40.02 | 15.9 | 112 | 54.08 | 65.8 |
| 68 | 40.36 | 16.7 | 113 | 54.39 | 67 |
| 69 | 40.69 | 17.6 | 114 | 54.69 | 68.1 |
| 70 | 41.03 | 18.5 | 115 | 55 | 69.2 |
| 71 | 41.36 | 19.4 | 116 | 55.31 | 70.2 |
| 72 | 41.7 | 20.3 | 117 | 55.62 | 71.3 |
| 73 | 42.03 | 21.3 | 118 | 55.94 | 72.4 |
| 74 | 42.35 | 22.2 | 119 | 56.25 | 73.4 |
| 75 | 42.68 | 23.2 | 120 | 56.56 | 74.4 |
| CAT-Q/SE total score | T-score | Percentile | CAT-Q/SE total score | T-score | Percentile |
| 121 | 56.88 | 75.4 | 141 | 63.81 | 91.6 |
| 122 | 57.2 | 76.4 | 142 | 64.21 | 92.2 |
| 123 | 57.52 | 77.4 | 143 | 64.61 | 92.8 |
| 124 | 57.84 | 78.4 | 144 | 65.03 | 93.4 |
| 125 | 58.17 | 79.3 | 145 | 65.45 | 93.9 |
| 126 | 58.49 | 80.2 | 146 | 65.89 | 94.4 |
| 127 | 58.82 | 81.1 | 147 | 66.34 | 94.9 |
| 128 | 59.15 | 82 | 148 | 66.81 | 95.4 |
| 129 | 59.49 | 82.9 | 149 | 67.29 | 95.8 |
| 130 | 59.82 | 83.7 | 150 | 67.8 | 96.2 |
| 131 | 60.16 | 84.5 | 151 | 68.32 | 96.7 |
| 132 | 60.51 | 85.3 | 152 | 68.88 | 97 |
| 133 | 60.86 | 86.1 | 153 | 69.47 | 97.4 |
| 134 | 61.21 | 86.9 | 154 | 70.11 | 97.8 |
| 135 | 61.56 | 87.6 | 155 | 70.81 | 98.1 |
| 136 | 61.92 | 88.3 | 156 | 71.6 | 98.5 |
| 137 | 62.29 | 89 | 157 | 72.53 | 98.8 |
| 138 | 62.66 | 89.7 | 158 | 73.76 | 99.1 |
| 139 | 63.04 | 90.4 | >158 | 75.76 | 99.5 |
| 140 | 63.42 | 91 |  |  |  |

**Supplementary Table 4.** CAT-Q/SE norms general population group, ages 10 to 15

| CAT-Q/SE total score | T-score | Percentile | CAT-Q/SE total score | T-score | Percentile |
| --- | --- | --- | --- | --- | --- |
| 25 | 26.54 | 0.9 | 70 | 45.64 | 33.1 |
| 26 | 26.97 | 1.1 | 71 | 46.06 | 34.7 |
| 27 | 27.39 | 1.2 | 72 | 46.48 | 36.3 |
| 28 | 27.82 | 1.3 | 73 | 46.91 | 37.9 |
| 29 | 28.24 | 1.5 | 74 | 47.33 | 39.5 |
| 30 | 28.67 | 1.6 | 75 | 47.75 | 41.1 |
| 31 | 29.09 | 1.8 | 76 | 48.18 | 42.8 |
| 32 | 29.52 | 2 | 77 | 48.6 | 44.4 |
| 33 | 29.94 | 2.2 | 78 | 49.02 | 46.1 |
| 34 | 30.37 | 2.5 | 79 | 49.45 | 47.8 |
| 35 | 30.79 | 2.7 | 80 | 49.87 | 49.5 |
| 36 | 31.22 | 3 | 81 | 50.29 | 51.2 |
| 37 | 31.64 | 3.3 | 82 | 50.72 | 52.9 |
| 38 | 32.07 | 3.6 | 83 | 51.14 | 54.5 |
| 39 | 32.49 | 4 | 84 | 51.56 | 56.2 |
| 40 | 32.92 | 4.4 | 85 | 51.99 | 57.9 |
| 41 | 33.34 | 4.8 | 86 | 52.41 | 59.5 |
| 42 | 33.76 | 5.2 | 87 | 52.83 | 61.2 |
| 43 | 34.19 | 5.7 | 88 | 53.26 | 62.8 |
| 44 | 34.61 | 6.2 | 89 | 53.68 | 64.4 |
| 45 | 35.04 | 6.7 | 90 | 54.1 | 65.9 |
| 46 | 35.46 | 7.3 | 91 | 54.52 | 67.5 |
| 47 | 35.89 | 7.9 | 92 | 54.95 | 69 |
| 48 | 36.31 | 8.6 | 93 | 55.37 | 70.4 |
| 49 | 36.74 | 9.2 | 94 | 55.79 | 71.9 |
| 50 | 37.16 | 10 | 95 | 56.21 | 73.3 |
| 51 | 37.58 | 10.7 | 96 | 56.64 | 74.7 |
| 52 | 38.01 | 11.5 | 97 | 57.06 | 76 |
| 53 | 38.43 | 12.4 | 98 | 57.48 | 77.3 |
| 54 | 38.86 | 13.3 | 99 | 57.91 | 78.5 |
| 55 | 39.28 | 14.2 | 100 | 58.33 | 79.8 |
| 56 | 39.7 | 15.2 | 101 | 58.75 | 80.9 |
| 57 | 40.13 | 16.2 | 102 | 59.17 | 82 |
| 58 | 40.55 | 17.2 | 103 | 59.6 | 83.1 |
| 59 | 40.98 | 18.3 | 104 | 60.02 | 84.2 |
| 60 | 41.4 | 19.5 | 105 | 60.44 | 85.2 |
| 61 | 41.82 | 20.7 | 106 | 60.86 | 86.1 |
| 62 | 42.25 | 21.9 | 107 | 61.28 | 87 |
| 63 | 42.67 | 23.2 | 108 | 61.71 | 87.9 |
| 64 | 43.1 | 24.5 | 109 | 62.13 | 88.7 |
| 65 | 43.52 | 25.8 | 110 | 62.55 | 89.5 |
| 66 | 43.94 | 27.2 | 111 | 62.97 | 90.3 |
| 67 | 44.37 | 28.7 | 112 | 63.39 | 91 |
| 68 | 44.79 | 30.1 | 113 | 63.82 | 91.6 |
| 69 | 45.21 | 31.6 | 114 | 64.24 | 92.3 |
| CAT-Q/SE total score | T-score | Percentile | CAT-Q/SE total score | T-score | Percentile |
| 115 | 64.66 | 92.9 | 132 | 71.83 | 98.5 |
| 116 | 65.08 | 93.4 | 133 | 72.25 | 98.7 |
| 117 | 65.5 | 93.9 | 134 | 72.67 | 98.8 |
| 118 | 65.93 | 94.4 | 135 | 73.09 | 99 |
| 119 | 66.35 | 94.9 | 136 | 73.51 | 99.1 |
| 120 | 66.77 | 95.3 | 137 | 73.94 | 99.2 |
| 121 | 67.19 | 95.7 | 138 | 74.36 | 99.3 |
| 122 | 67.61 | 96.1 | 139 | 74.78 | 99.3 |
| 123 | 68.04 | 96.4 | 140 | 75.2 | 99.4 |
| 124 | 68.46 | 96.8 | 141 | 75.62 | 99.5 |
| 125 | 68.88 | 97 | 142 | 76.04 | 99.5 |
| 126 | 69.3 | 97.3 | 143 | 76.46 | 99.6 |
| 127 | 69.72 | 97.6 | 144 | 76.88 | 99.6 |
| 128 | 70.14 | 97.8 | 145 | 77.3 | 99.7 |
| 129 | 70.56 | 98 | 146 | 77.72 | 99.7 |
| 130 | 70.99 | 98.2 | >146 | 77.82 | 99.7 |
| 131 | 71.41 | 98.4 |  |  |  |

**Supplementary Table 5.** CAT-Q/SE norms general population group, ages 15 to 20

| CAT-Q/SE total score | T-score | Percentile | CAT-Q/SE total score | T-score | Percentile |
| --- | --- | --- | --- | --- | --- |
| 25 | 24.77 | 0.6 | 70 | 42.51 | 22.7 |
| 26 | 25.16 | 0.6 | 71 | 42.9 | 23.9 |
| 27 | 25.56 | 0.7 | 72 | 43.3 | 25.1 |
| 28 | 25.95 | 0.8 | 73 | 43.69 | 26.4 |
| 29 | 26.35 | 0.9 | 74 | 44.08 | 27.7 |
| 30 | 26.75 | 1 | 75 | 44.47 | 29 |
| 31 | 27.14 | 1.1 | 76 | 44.86 | 30.4 |
| 32 | 27.54 | 1.2 | 77 | 45.25 | 31.8 |
| 33 | 27.93 | 1.4 | 78 | 45.65 | 33.2 |
| 34 | 28.33 | 1.5 | 79 | 46.04 | 34.6 |
| 35 | 28.73 | 1.7 | 80 | 46.43 | 36 |
| 36 | 29.12 | 1.8 | 81 | 46.82 | 37.5 |
| 37 | 29.52 | 2 | 82 | 47.21 | 39 |
| 38 | 29.91 | 2.2 | 83 | 47.6 | 40.5 |
| 39 | 30.31 | 2.4 | 84 | 47.99 | 42 |
| 40 | 30.7 | 2.7 | 85 | 48.38 | 43.6 |
| 41 | 31.1 | 2.9 | 86 | 48.77 | 45.1 |
| 42 | 31.49 | 3.2 | 87 | 49.16 | 46.7 |
| 43 | 31.89 | 3.5 | 88 | 49.55 | 48.2 |
| 44 | 32.28 | 3.8 | 89 | 49.95 | 49.8 |
| 45 | 32.68 | 4.2 | 90 | 50.34 | 51.3 |
| 46 | 33.07 | 4.5 | 91 | 50.73 | 52.9 |
| 47 | 33.47 | 4.9 | 92 | 51.12 | 54.4 |
| 48 | 33.86 | 5.3 | 93 | 51.51 | 56 |
| 49 | 34.25 | 5.8 | 94 | 51.9 | 57.5 |
| 50 | 34.65 | 6.2 | 95 | 52.29 | 59 |
| 51 | 35.04 | 6.7 | 96 | 52.68 | 60.5 |
| 52 | 35.44 | 7.3 | 97 | 53.06 | 62 |
| 53 | 35.83 | 7.8 | 98 | 53.45 | 63.5 |
| 54 | 36.22 | 8.4 | 99 | 53.84 | 65 |
| 55 | 36.62 | 9 | 100 | 54.23 | 66.4 |
| 56 | 37.01 | 9.7 | 101 | 54.62 | 67.8 |
| 57 | 37.4 | 10.4 | 102 | 55.01 | 69.2 |
| 58 | 37.8 | 11.1 | 103 | 55.4 | 70.5 |
| 59 | 38.19 | 11.9 | 104 | 55.79 | 71.9 |
| 60 | 38.58 | 12.7 | 105 | 56.18 | 73.2 |
| 61 | 38.98 | 13.5 | 106 | 56.57 | 74.4 |
| 62 | 39.37 | 14.4 | 107 | 56.96 | 75.7 |
| 63 | 39.76 | 15.3 | 108 | 57.35 | 76.9 |
| 64 | 40.16 | 16.2 | 109 | 57.73 | 78 |
| 65 | 40.55 | 17.2 | 110 | 58.12 | 79.2 |
| 66 | 40.94 | 18.3 | 111 | 58.51 | 80.3 |
| 67 | 41.33 | 19.3 | 112 | 58.9 | 81.3 |
| 68 | 41.73 | 20.4 | 113 | 59.29 | 82.3 |
| 69 | 42.12 | 21.5 | 114 | 59.68 | 83.3 |
| CAT-Q/SE total score | T-score | Percentile | CAT-Q/SE total score | T-score | Percentile |
| 115 | 60.06 | 84.3 | 139 | 69.35 | 97.3 |
| 116 | 60.45 | 85.2 | 140 | 69.73 | 97.6 |
| 117 | 60.84 | 86.1 | 141 | 70.12 | 97.8 |
| 118 | 61.23 | 86.9 | 142 | 70.5 | 98 |
| 119 | 61.61 | 87.7 | 143 | 70.89 | 98.2 |
| 120 | 62 | 88.5 | 144 | 71.27 | 98.3 |
| 121 | 62.39 | 89.2 | 145 | 71.66 | 98.5 |
| 122 | 62.78 | 89.9 | 146 | 72.05 | 98.6 |
| 123 | 63.16 | 90.6 | 147 | 72.43 | 98.8 |
| 124 | 63.55 | 91.2 | 148 | 72.82 | 98.9 |
| 125 | 63.94 | 91.8 | 149 | 73.2 | 99 |
| 126 | 64.32 | 92.4 | 150 | 73.59 | 99.1 |
| 127 | 64.71 | 92.9 | 151 | 73.97 | 99.2 |
| 128 | 65.1 | 93.4 | 152 | 74.35 | 99.3 |
| 129 | 65.49 | 93.9 | 153 | 74.74 | 99.3 |
| 130 | 65.87 | 94.4 | 154 | 75.12 | 99.4 |
| 131 | 66.26 | 94.8 | 155 | 75.51 | 99.5 |
| 132 | 66.64 | 95.2 | 156 | 75.89 | 99.5 |
| 133 | 67.03 | 95.6 | 157 | 76.28 | 99.6 |
| 134 | 67.42 | 95.9 | 158 | 76.66 | 99.6 |
| 135 | 67.8 | 96.2 | 159 | 77.04 | 99.7 |
| 136 | 68.19 | 96.6 | 160 | 77.43 | 99.7 |
| 137 | 68.58 | 96.8 | 161 | 77.81 | 99.7 |
| 138 | 68.96 | 97.1 | >161 | 77.82 | 99.7 |

**Supplementary Table 6.** CAT-Q/SE norms general population group, ages 20 to 40

| CAT-Q/SE total score | T-score | Percentile | CAT-Q/SE total score | T-score | Percentile |
| --- | --- | --- | --- | --- | --- |
| 25 | 24.18 | 0.5 | 70 | 41.43 | 19.6 |
| 26 | 24.56 | 0.5 | 71 | 41.81 | 20.6 |
| 27 | 24.95 | 0.6 | 72 | 42.19 | 21.7 |
| 28 | 25.34 | 0.7 | 73 | 42.57 | 22.9 |
| 29 | 25.73 | 0.8 | 74 | 42.94 | 24 |
| 30 | 26.11 | 0.8 | 75 | 43.32 | 25.2 |
| 31 | 26.5 | 0.9 | 76 | 43.7 | 26.4 |
| 32 | 26.89 | 1 | 77 | 44.08 | 27.7 |
| 33 | 27.28 | 1.2 | 78 | 44.45 | 29 |
| 34 | 27.66 | 1.3 | 79 | 44.83 | 30.3 |
| 35 | 28.05 | 1.4 | 80 | 45.21 | 31.6 |
| 36 | 28.43 | 1.6 | 81 | 45.58 | 32.9 |
| 37 | 28.82 | 1.7 | 82 | 45.96 | 34.3 |
| 38 | 29.21 | 1.9 | 83 | 46.34 | 35.7 |
| 39 | 29.59 | 2.1 | 84 | 46.71 | 37.1 |
| 40 | 29.98 | 2.3 | 85 | 47.09 | 38.5 |
| 41 | 30.36 | 2.5 | 86 | 47.46 | 40 |
| 42 | 30.75 | 2.7 | 87 | 47.84 | 41.5 |
| 43 | 31.13 | 3 | 88 | 48.22 | 42.9 |
| 44 | 31.51 | 3.2 | 89 | 48.59 | 44.4 |
| 45 | 31.9 | 3.5 | 90 | 48.97 | 45.9 |
| 46 | 32.28 | 3.8 | 91 | 49.34 | 47.4 |
| 47 | 32.67 | 4.2 | 92 | 49.71 | 48.9 |
| 48 | 33.05 | 4.5 | 93 | 50.09 | 50.4 |
| 49 | 33.43 | 4.9 | 94 | 50.46 | 51.8 |
| 50 | 33.82 | 5.3 | 95 | 50.84 | 53.3 |
| 51 | 34.2 | 5.7 | 96 | 51.21 | 54.8 |
| 52 | 34.58 | 6.2 | 97 | 51.58 | 56.3 |
| 53 | 34.96 | 6.6 | 98 | 51.96 | 57.8 |
| 54 | 35.34 | 7.1 | 99 | 52.33 | 59.2 |
| 55 | 35.73 | 7.7 | 100 | 52.7 | 60.7 |
| 56 | 36.11 | 8.2 | 101 | 53.08 | 62.1 |
| 57 | 36.49 | 8.8 | 102 | 53.45 | 63.5 |
| 58 | 36.87 | 9.5 | 103 | 53.82 | 64.9 |
| 59 | 37.25 | 10.1 | 104 | 54.19 | 66.2 |
| 60 | 37.63 | 10.8 | 105 | 54.56 | 67.6 |
| 61 | 38.01 | 11.5 | 106 | 54.94 | 68.9 |
| 62 | 38.39 | 12.3 | 107 | 55.31 | 70.2 |
| 63 | 38.77 | 13.1 | 108 | 55.68 | 71.5 |
| 64 | 39.15 | 13.9 | 109 | 56.05 | 72.7 |
| 65 | 39.53 | 14.8 | 110 | 56.42 | 74 |
| 66 | 39.91 | 15.7 | 111 | 56.79 | 75.1 |
| 67 | 40.29 | 16.6 | 112 | 57.16 | 76.3 |
| 68 | 40.67 | 17.5 | 113 | 57.53 | 77.4 |
| 69 | 41.05 | 18.5 | 114 | 57.9 | 78.5 |
| CAT-Q/SE total score | T-score | Percentile | CAT-Q/SE total score | T-score | Percentile |
| 115 | 58.27 | 79.6 | 143 | 68.56 | 96.8 |
| 116 | 58.64 | 80.6 | 144 | 68.92 | 97.1 |
| 117 | 59.01 | 81.6 | 145 | 69.29 | 97.3 |
| 118 | 59.38 | 82.6 | 146 | 69.65 | 97.5 |
| 119 | 59.75 | 83.5 | 147 | 70.01 | 97.7 |
| 120 | 60.12 | 84.4 | 148 | 70.38 | 97.9 |
| 121 | 60.49 | 85.3 | 149 | 70.74 | 98.1 |
| 122 | 60.86 | 86.1 | 150 | 71.1 | 98.3 |
| 123 | 61.23 | 86.9 | 151 | 71.47 | 98.4 |
| 124 | 61.59 | 87.7 | 152 | 71.83 | 98.5 |
| 125 | 61.96 | 88.4 | 153 | 72.19 | 98.7 |
| 126 | 62.33 | 89.1 | 154 | 72.56 | 98.8 |
| 127 | 62.7 | 89.8 | 155 | 72.92 | 98.9 |
| 128 | 63.07 | 90.4 | 156 | 73.28 | 99 |
| 129 | 63.43 | 91 | 157 | 73.64 | 99.1 |
| 130 | 63.8 | 91.6 | 158 | 74.01 | 99.2 |
| 131 | 64.17 | 92.2 | 159 | 74.37 | 99.3 |
| 132 | 64.53 | 92.7 | 160 | 74.73 | 99.3 |
| 133 | 64.9 | 93.2 | 161 | 75.09 | 99.4 |
| 134 | 65.27 | 93.7 | 162 | 75.45 | 99.5 |
| 135 | 65.63 | 94.1 | 163 | 75.81 | 99.5 |
| 136 | 66 | 94.5 | 164 | 76.18 | 99.6 |
| 137 | 66.37 | 94.9 | 165 | 76.54 | 99.6 |
| 138 | 66.73 | 95.3 | 166 | 76.9 | 99.6 |
| 139 | 67.1 | 95.6 | 167 | 77.26 | 99.7 |
| 140 | 67.46 | 96 | 168 | 77.62 | 99.7 |
| 141 | 67.83 | 96.3 | >168 | 77.82 | 99.7 |
| 142 | 68.19 | 96.6 |  |  |  |

**Supplementary Table 7.** CAT-Q/SE norms general population group, ages 40 to 60

| CAT-Q/SE total score | T-score | Percentile | CAT-Q/SE total score | T-score | Percentile |
| --- | --- | --- | --- | --- | --- |
| 25 | 28.78 | 1.7 | 70 | 48.77 | 45.1 |
| 26 | 29.29 | 1.9 | 71 | 49.16 | 46.7 |
| 27 | 29.79 | 2.2 | 72 | 49.55 | 48.2 |
| 28 | 30.3 | 2.4 | 73 | 49.94 | 49.8 |
| 29 | 30.79 | 2.7 | 74 | 50.32 | 51.3 |
| 30 | 31.29 | 3.1 | 75 | 50.71 | 52.8 |
| 31 | 31.78 | 3.4 | 76 | 51.09 | 54.3 |
| 32 | 32.27 | 3.8 | 77 | 51.47 | 55.9 |
| 33 | 32.75 | 4.2 | 78 | 51.85 | 57.3 |
| 34 | 33.24 | 4.7 | 79 | 52.23 | 58.8 |
| 35 | 33.71 | 5.2 | 80 | 52.6 | 60.3 |
| 36 | 34.19 | 5.7 | 81 | 52.98 | 61.7 |
| 37 | 34.66 | 6.3 | 82 | 53.35 | 63.1 |
| 38 | 35.13 | 6.8 | 83 | 53.72 | 64.5 |
| 39 | 35.59 | 7.5 | 84 | 54.09 | 65.9 |
| 40 | 36.06 | 8.2 | 85 | 54.46 | 67.2 |
| 41 | 36.52 | 8.9 | 86 | 54.83 | 68.5 |
| 42 | 36.97 | 9.6 | 87 | 55.19 | 69.8 |
| 43 | 37.43 | 10.4 | 88 | 55.56 | 71.1 |
| 44 | 37.88 | 11.3 | 89 | 55.92 | 72.3 |
| 45 | 38.33 | 12.2 | 90 | 56.28 | 73.5 |
| 46 | 38.77 | 13.1 | 91 | 56.64 | 74.7 |
| 47 | 39.22 | 14 | 92 | 57 | 75.8 |
| 48 | 39.66 | 15 | 93 | 57.36 | 76.9 |
| 49 | 40.09 | 16.1 | 94 | 57.71 | 78 |
| 50 | 40.53 | 17.2 | 95 | 58.06 | 79 |
| 51 | 40.96 | 18.3 | 96 | 58.42 | 80 |
| 52 | 41.39 | 19.5 | 97 | 58.77 | 81 |
| 53 | 41.82 | 20.7 | 98 | 59.12 | 81.9 |
| 54 | 42.25 | 21.9 | 99 | 59.47 | 82.8 |
| 55 | 42.67 | 23.2 | 100 | 59.82 | 83.7 |
| 56 | 43.09 | 24.5 | 101 | 60.16 | 84.5 |
| 57 | 43.51 | 25.8 | 102 | 60.51 | 85.3 |
| 58 | 43.93 | 27.2 | 103 | 60.85 | 86.1 |
| 59 | 44.34 | 28.6 | 104 | 61.19 | 86.9 |
| 60 | 44.75 | 30 | 105 | 61.54 | 87.6 |
| 61 | 45.16 | 31.4 | 106 | 61.88 | 88.3 |
| 62 | 45.57 | 32.9 | 107 | 62.22 | 88.9 |
| 63 | 45.98 | 34.4 | 108 | 62.55 | 89.5 |
| 64 | 46.38 | 35.9 | 109 | 62.89 | 90.1 |
| 65 | 46.79 | 37.4 | 110 | 63.23 | 90.7 |
| 66 | 47.19 | 38.9 | 111 | 63.56 | 91.2 |
| 67 | 47.59 | 40.5 | 112 | 63.89 | 91.8 |
| 68 | 47.98 | 42 | 113 | 64.23 | 92.3 |
| 69 | 48.38 | 43.6 | 114 | 64.56 | 92.7 |
| CAT-Q/SE total score | T-score | Percentile | CAT-Q/SE total score | T-score | Percentile |
| 115 | 64.89 | 93.2 | 137 | 71.9 | 98.6 |
| 116 | 65.22 | 93.6 | 138 | 72.2 | 98.7 |
| 117 | 65.54 | 94 | 139 | 72.51 | 98.8 |
| 118 | 65.87 | 94.4 | 140 | 72.82 | 98.9 |
| 119 | 66.2 | 94.7 | 141 | 73.12 | 99 |
| 120 | 66.52 | 95.1 | 142 | 73.43 | 99 |
| 121 | 66.85 | 95.4 | 143 | 73.73 | 99.1 |
| 122 | 67.17 | 95.7 | 144 | 74.03 | 99.2 |
| 123 | 67.49 | 96 | 145 | 74.34 | 99.3 |
| 124 | 67.81 | 96.3 | 146 | 74.64 | 99.3 |
| 125 | 68.13 | 96.5 | 147 | 74.94 | 99.4 |
| 126 | 68.45 | 96.7 | 148 | 75.24 | 99.4 |
| 127 | 68.77 | 97 | 149 | 75.54 | 99.5 |
| 128 | 69.09 | 97.2 | 150 | 75.83 | 99.5 |
| 129 | 69.4 | 97.4 | 151 | 76.13 | 99.6 |
| 130 | 69.72 | 97.6 | 152 | 76.43 | 99.6 |
| 131 | 70.03 | 97.7 | 153 | 76.72 | 99.6 |
| 132 | 70.34 | 97.9 | 154 | 77.02 | 99.7 |
| 133 | 70.66 | 98.1 | 155 | 77.31 | 99.7 |
| 134 | 70.97 | 98.2 | 156 | 77.6 | 99.7 |
| 135 | 71.28 | 98.3 | >156 | 77.82 | 99.7 |
| 136 | 71.59 | 98.5 |  |  |  |

**Supplementary Table 8.** CAT-Q/SE norms general population group, ages 60 or older

| CAT-Q/SE total score | T-score | Percentile | CAT-Q/SE total score | T-score | Percentile |
| --- | --- | --- | --- | --- | --- |
| 25 | 28.34 | 1.5 | 70 | 55.27 | 70.1 |
| 26 | 30.68 | 2.7 | 71 | 55.6 | 71.2 |
| 27 | 32.28 | 3.8 | 72 | 55.92 | 72.3 |
| 28 | 33.57 | 5 | 73 | 56.24 | 73.4 |
| 29 | 34.69 | 6.3 | 74 | 56.56 | 74.4 |
| 30 | 35.69 | 7.6 | 75 | 56.87 | 75.4 |
| 31 | 36.6 | 9 | 76 | 57.18 | 76.4 |
| 32 | 37.44 | 10.5 | 77 | 57.49 | 77.3 |
| 33 | 38.23 | 12 | 78 | 57.8 | 78.2 |
| 34 | 38.97 | 13.5 | 79 | 58.1 | 79.1 |
| 35 | 39.68 | 15.1 | 80 | 58.4 | 80 |
| 36 | 40.35 | 16.7 | 81 | 58.7 | 80.8 |
| 37 | 40.99 | 18.4 | 82 | 58.99 | 81.6 |
| 38 | 41.61 | 20.1 | 83 | 59.28 | 82.3 |
| 39 | 42.2 | 21.8 | 84 | 59.57 | 83.1 |
| 40 | 42.78 | 23.5 | 85 | 59.86 | 83.8 |
| 41 | 43.33 | 25.2 | 86 | 60.15 | 84.5 |
| 42 | 43.87 | 27 | 87 | 60.43 | 85.1 |
| 43 | 44.4 | 28.8 | 88 | 60.71 | 85.8 |
| 44 | 44.91 | 30.5 | 89 | 60.99 | 86.4 |
| 45 | 45.4 | 32.3 | 90 | 61.26 | 87 |
| 46 | 45.89 | 34.1 | 91 | 61.54 | 87.6 |
| 47 | 46.36 | 35.8 | 92 | 61.81 | 88.1 |
| 48 | 46.83 | 37.6 | 93 | 62.08 | 88.6 |
| 49 | 47.28 | 39.3 | 94 | 62.34 | 89.1 |
| 50 | 47.73 | 41 | 95 | 62.61 | 89.6 |
| 51 | 48.16 | 42.7 | 96 | 62.87 | 90.1 |
| 52 | 48.59 | 44.4 | 97 | 63.14 | 90.6 |
| 53 | 49.01 | 46.1 | 98 | 63.4 | 91 |
| 54 | 49.42 | 47.7 | 99 | 63.66 | 91.4 |
| 55 | 49.83 | 49.3 | 100 | 63.91 | 91.8 |
| 56 | 50.23 | 50.9 | 101 | 64.17 | 92.2 |
| 57 | 50.62 | 52.5 | 102 | 64.42 | 92.5 |
| 58 | 51.01 | 54 | 103 | 64.67 | 92.9 |
| 59 | 51.39 | 55.5 | 104 | 64.92 | 93.2 |
| 60 | 51.77 | 57 | 105 | 65.17 | 93.5 |
| 61 | 52.14 | 58.5 | 106 | 65.42 | 93.8 |
| 62 | 52.5 | 59.9 | 107 | 65.66 | 94.1 |
| 63 | 52.86 | 61.3 | 108 | 65.91 | 94.4 |
| 64 | 53.22 | 62.6 | 109 | 66.15 | 94.7 |
| 65 | 53.57 | 64 | 110 | 66.39 | 94.9 |
| 66 | 53.92 | 65.2 | 111 | 66.63 | 95.2 |
| 67 | 54.26 | 66.5 | 112 | 66.87 | 95.4 |
| 68 | 54.6 | 67.7 | 113 | 67.11 | 95.6 |
| 69 | 54.94 | 68.9 | 114 | 67.34 | 95.9 |
| CAT-Q/SE total score | T-score | Percentile | CAT-Q/SE total score | T-score | Percentile |
| 115 | 67.58 | 96.1 | 141 | 73.27 | 99 |
| 116 | 67.81 | 96.3 | 142 | 73.47 | 99.1 |
| 117 | 68.04 | 96.4 | 143 | 73.68 | 99.1 |
| 118 | 68.27 | 96.6 | 144 | 73.88 | 99.2 |
| 119 | 68.5 | 96.8 | 145 | 74.09 | 99.2 |
| 120 | 68.73 | 96.9 | 146 | 74.29 | 99.2 |
| 121 | 68.96 | 97.1 | 147 | 74.49 | 99.3 |
| 122 | 69.18 | 97.2 | 148 | 74.69 | 99.3 |
| 123 | 69.41 | 97.4 | 149 | 74.89 | 99.4 |
| 124 | 69.63 | 97.5 | 150 | 75.09 | 99.4 |
| 125 | 69.85 | 97.6 | 151 | 75.29 | 99.4 |
| 126 | 70.07 | 97.8 | 152 | 75.48 | 99.5 |
| 127 | 70.29 | 97.9 | 153 | 75.68 | 99.5 |
| 128 | 70.51 | 98 | 154 | 75.88 | 99.5 |
| 129 | 70.73 | 98.1 | 155 | 76.07 | 99.5 |
| 130 | 70.95 | 98.2 | 156 | 76.27 | 99.6 |
| 131 | 71.16 | 98.3 | 157 | 76.46 | 99.6 |
| 132 | 71.38 | 98.4 | 158 | 76.65 | 99.6 |
| 133 | 71.59 | 98.5 | 159 | 76.84 | 99.6 |
| 134 | 71.81 | 98.5 | 160 | 77.03 | 99.7 |
| 135 | 72.02 | 98.6 | 161 | 77.23 | 99.7 |
| 136 | 72.23 | 98.7 | 162 | 77.42 | 99.7 |
| 137 | 72.44 | 98.8 | 163 | 77.6 | 99.7 |
| 138 | 72.65 | 98.8 | 164 | 77.79 | 99.7 |
| 139 | 72.86 | 98.9 | >164 | 77.82 | 99.7 |
| 140 | 73.06 | 98.9 |  |  |  |
